# Preparative Separation of Three Monoterpenes from* Perilla frutescens* var.* crispa* Using Centrifugal Partition Chromatography

**DOI:** 10.1155/2019/8751345

**Published:** 2019-01-09

**Authors:** Bomi Nam, Sunil Babu Paudel, Jin-Baek Kim, Chang Hyun Jin, Dongho Lee, Joo-Won Nam, Ah-Reum Han

**Affiliations:** ^1^Advanced Radiation Technology Institute, Korea Atomic Energy Research Institute, Jeongeup-si, Jeollabuk-do 56212, Republic of Korea; ^2^College of Pharmacy, Yeungnam University, Gyeongsan-si, Gyeongsangbuk-do 38541, Republic of Korea; ^3^Department of Biosystems and Biotechnology, Korea University, Seoul 02841, Republic of Korea

## Abstract

Three monoterpenes, namely, 9-hydroxy isoegomaketone (**1**), isoegomaketone (**2**), and perilla ketone (**3**), were successfully separated from the supercritical carbon dioxide (SC-CO_2_) extract of the leaves of* Perilla frutescens* var.* crispa* (cv. Antisperill; Lamiaceae) by centrifugal partition chromatography (CPC). To obtain large quantities of these materials required for studies on their mechanism of action and* in vivo* effectiveness in inflammation, we used CPC because of its high loading capacity and reproducibility to purify the three compounds. Compound** 1** (2.60 mg, 96.7% purity at 254 nm) was purified from 500 mg of the SC-CO_2_ extract of* P. frutescens* var.* crispa* (cv. Antisperill), using a two-phase solvent system comprising *n*-hexane/ethyl acetate/ethanol/water (5:5:5:5 v/v) in a descending mode. As compounds** 2** (56.1 mg, 97.6% purity at 254 nm) and** 3** (78.6 mg, 96.1% purity at 254 nm) are highly volatile and difficult to recover from an aqueous mobile phase after purification during the drying process, they were obtained from the same amount of the processed extract in an ascending mode using the upper organic phase as the mobile phase (*n*-hexane/ethyl acetate/ethanol/water, 8:2:8:2 v/v). The structures of compounds** 1**–**3** were confirmed by ^1^H- and ^13^C-NMR analysis. Thus, based on our findings, we recommend centrifugal partition chromatography as a powerful technique for purifying the active principal compounds** 1** and** 2** from the leaves of* P. frutescens* var.* crispa*.

## 1. Introduction

**Figure 1:**
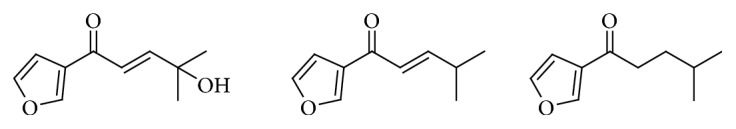
Chemical structures of compounds** 1**–**3** extracted from the leaves of* P. frutescens* var.* crispa* (cv. Antisperill).

*Perilla frutescens* var.* crispa* (Lamiaceae) is widely distributed throughout Asia, and its leaves are used both as a vegetable and as a traditional medicine to treat indigestion, gastritis, and sea-food poisoning [1]. The ethanol or supercritical carbon dioxide (SC-CO_2_) extract of* P. frutescens* var.* crispa* and its constituents including monoterpenes, flavonoids, and phenolic acids, exhibit diverse biological effects, such as antioxidant [2, 3], anti-inflammatory [4–9], and antitumor [10, 11] effects. A new cultivar of this plant, namely,* P. frutescens* var.* crispa *(cv. Antisperill), was developed by a gamma-irradiated mutation breeding of the original plant* P. frutescens* var.* crispa* [7]. In our previous studies on quantitative analyses of the components of this new cultivars [8, 9], the SC-CO_2_ extract of this new cultivar possesses higher content of monoterpenes, 9-hydroxy isoegomaketone (**1**; 1.33 ± 0.07 mg/g, dry w/w), isoegomaketone (**2**; 2.76 ± 0.05 mg/g, dry w/w), and perilla ketone (**3**; 6.96 ± 0.17 mg/g, dry w/w), compared with their contents of the SC-CO_2_ extract of the original cultivar (Figure 1). In addition, in our previous biological evaluations on these monoterpenes, compounds** 1** and** 2** inhibited nitric oxide (NO) production in lipopolysaccharide- (LPS-) stimulated macrophages [4, 11]. Furthermore, compound** 2** downregulated inducible NO synthase protein levels in LPS-stimulated RAW 264.7 macrophages through heme oxygenase-1 induction and interferon-*β*/STAT-1 pathway inhibition [4] and enhanced the development of collagen antibody-induced arthritis in male Balb/c mice [12]. Based on these evidences,** 1** and** 2** were identified as the anti-inflammatory principal components and the anti-inflammatory of the SC-CO_2_ extract of a new cultivar with higher contents of these two components was greater than that of the SC-CO_2_ extract of the original cultivar [8]. For developing health functional food and/or botanical drug product using the SC-CO_2_ extract of the leaves of* P. frutescens* var.* crispa *(cv. Antisperill), large amounts of purified compounds are required to evaluate the* in vivo* and* in vitro* anti-inflammatory potential of this plant. For this purpose, we developed an efficient method for the preparative isolation of these compounds.

Countercurrent separation (CCS) was designed to perform chromatography using a liquid-liquid biphasic system without solid support to maintain the stationary phase [13]. Centrifugal partition chromatography (CPC) is a form of CCS operated by hydrostatic force created by the centrifugal field in the rotor of a one-axis centrifuge [14]. As CCS offers the advantages of no irreversible adsorption or denaturation of the sample, simple column regeneration, 100% theoretical recovery/yield, and the enhancement of sample-loading capacity, it is a popular technique for scaled-up separations with minimal sample preparation and isolation of multiple target compounds in a single step [13, 14]. Thus, this technique has been widely used for preparative separation and purification of natural products [13, 15].

In our previous phytochemical studies on the extract of the leaves of* P. frutescens* var.* crispa *(cv. Antisperill) using column chromatography [9], there was a problem that this extract contained a large amount of chlorophylls, resulting in the time-consuming separation steps and a low yield of pure compounds. In addition, although several previous studies have demonstrated the separation of phenolic acids [16, 17], flavonoids [16], anthocyanins [18], and a monoterpene, perilla ketone [19] from* Perilla* species, there has no report on the separation and purification of** 1** and** 2** from* Perilla *species using CCS. Therefore, the present study aimed to establish an efficient CPC method for the purification of three monoterpenes, compounds** 1**–**3**, from the SC-CO_2_ extract of the leaves of* P. frutescens* var.* crispa *(cv. Antisperill).

## 2. Materials and Methods

### 2.1. General

CPC was performed on the Gilson CPC 250 system (Gilson Inc., Middleton, WI, USA) equipped with a 250 mL rotor, a 10 mL sample loop, a Shimadzu LC-8A pump (Shimadzu, Kyoto, Japan), and a Shimadzu SPD-10A UV/Vis detector. The equipment used for HPLC analysis was the Agilent 1200 system (Agilent Technologies Co., Santa Clara, CA, USA) equipped with a YMC-Triart C18 column (5 *μ*m, 250 mm × 4.6 mm; YMC Co.) and the ChemStation software. The NMR experiment was performed on the JNM-ECA 500 MHz NMR instrument (JEOL Ltd., Tokyo, Japan) with tetramethylsilane as an internal standard. All other chemicals and solvents used in this study were of analytical grade.

### 2.2. Plant Material

*P. frutescens* var.* crispa* (cv. Antisperill) was developed by gamma irradiating (200 Gy) the seeds of the original plant* P. frutescens* var.* crispa* using a labeled Cobalt (^60^Co) source, followed by selection based on the screening of anti-inflammatory activity and active compound concentrations as well as the examination of stable inheritance of phenotype for 3 years (1995–1998) at the Advanced Radiation Technology Institute, Korea Atomic Energy Research Institute (Jeongeup-si, Jeollabuk-do, Korea). The leaves of* P. frutescens* var.* crispa* (cv. Antisperill) were collected each year shortly before the flowering period. The seeds of this plant have been deposited for patent processing in the Korean Collection for Type Cultures, Biological Resource Center, Korea Research Institute of Bioscience and Biotechnology (August 2016). The voucher specimens have been deposited at the Advanced Radiation Technology Institute, Korea Atomic Energy Research Institute (Jeongeup-si, Jeollabuk-do, Korea).

### 2.3. Preparation of Crude Sample

The dried leaves of* P. frutescens* var.* crispa* (cv. Antisperill) (45 kg) were pulverized and then prepared by the SC-CO_2_ extraction method using the supercritical fluid extraction system (SCFE-P100; Ilshin Autoclave Co., Daejeon, Korea). The powdered sample was placed into the extraction column of the SC-CO_2_ extractor. The predetermined conditions for the procedure were as follows: pressure, 400 bar; temperature, 50°C, CO_2_ flow rate (99.9%), constant at 3 L/min; and extraction time, 4 h. The resultant oil was collected (480 g; 1.92% w/w) and stored in a refrigerator at 4°C.

### 2.4. Evaluation of Partition Coefficient (K) and Separation Factor (*α*)

The two-phase solvent system was selected according to the partition coefficient (*K*) of the target compounds in the crude sample. The *K* value was defined as the peak area of the target compound in the stationary phase divided by that in the mobile phase [20]. The *K* value was determined using HPLC as follows: 1-mg crude sample was added to a 1.5-mL tube, and then, 500 *μ*L of each phase of the preequilibrated two-phase solvent system was added and vigorously shaken. After the two-phase samples were thoroughly equilibrated, 200 *μ*L of each phase was collected and subjected to HPLC analysis.

The separation factor (*α*) value was the ratio of the two* K* values and was obtained by dividing the* K* values of the two compounds (*α* =* K*_1_/*K*_2_, where* K*_1_ >* K*_2_). The values were recommended to be >1.5 [20].

### 2.5. Preparation of the Two-Phase Solvent System and Sample Solution

In this study, two different biphasic solvent systems were used in the orthogonal operational mode: *n*-hexane/ethyl acetate/ethanol/water (5:5:5:5, v/v) in the descending mode and *n*-hexane/ethyl acetate/ethanol/water (8:2:8:2, v/v) in the ascending mode. Each solvent was added to a separation funnel and shaken thoroughly. After equilibration, the upper and lower phases were separated and degassed by sonication for 30 min before use. For preparing the injection, 500-mg crude sample was dissolved in 2.5 mL of each phase.

### 2.6. CPC Separation Procedure

Depending on the density of the phase used, CPC has two operation modes. If the lower phase is used as the mobile phase, the descending mode should be selected. This operation mode is believed to provide more stable retention of the stationary phase, although it takes longer time for drying fractions. On the other hand, if the upper phase is used as the mobile phase, CPC should be operated in the ascending mode. In this mode, solvents can be easily evaporated from the collected fractions [20]. In this experiment, a descending mode was used for purifying compound** 1** and an ascending mode was used to obtain highly volatile compounds** 2** and** 3**. The column was first filled with the stationary phase at the flow rate of 10 mL/min with rotation speed of 500 rpm, and then, the mobile phase was pumped into the column at the same flow rate while the instrument was run at the revolution rate of 1600 rpm. After the mobile phase was flowed out of the column and a hydrostatic equilibrium was established in the column, the prepared sample solution was injected. The fractions were collected manually and monitored using a UV detector at 254 nm. Reproducible results were obtained from three repeated CPC experiments.

### 2.7. HPLC-DAD-ESI/MS Analysis of Purified Compounds

The SC-CO_2_ extract and each peak fraction from CPC were weighed accurately and dissolved in MeOH at 1.0 and 0.5 mg/mL, respectively, and filtered through a syringe filter (0.45 *μ*m) for HPLC analysis. The HPLC analysis was performed using an YMC-Triart C18 column (5 *μ*m, 250 × 4.6 mm; YMC Co., Kyoto, Japan) with a gradient solvent system of acetonitrile and water (45:55–55:45). The flow rate was maintained at 0.8 mL/min, and the injection volume was set to 10 *μ*L. Chromatograms were acquired at 254 nm using the DAD detector. The mass spectra were measured between* m/z* 100 and 1000 in the positive ionization mode (ESI^+^) at the scan rate of 1.06 s/cycle and was monitored using a diode array detector. The mass spectrometric conditions were as follows: capillary voltage = 4000 V; drying gas flow = 10 L/min (N_2_); nebulizer pressure = 30 psi; and drying gas temperature = 350°C.

## 3. Results and Discussion

### 3.1. Selection of the Two-Phase Solvent System

**Figure 2:**
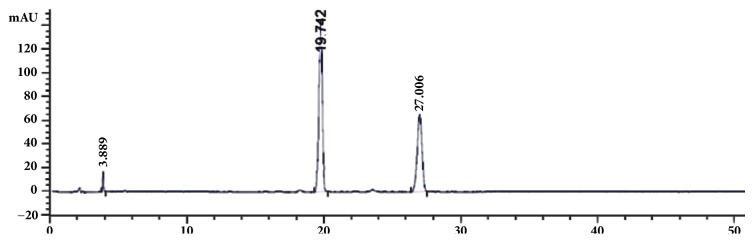
HPLC profile of the SC-CO_2_ extract of the leaves of* P. frutescens* var.* crispa* (cv. Antisperill) at 254 nm. Peak 1: 9-hydroxy isoegomaketone (**1**); peak 2: isoegomaketone (**2**); peak 3: perilla ketone (**3**) (for chromatography conditions, see Section 2).

The SC-CO_2_ extract of the leaves of* P. frutescens* var.* crispa* (cv. Antisperill) mainly comprised three major compounds, namely 9-hydroxy isoegomaketone (**1**), isoegomaketone (**2**), and peril ketone (**3**), as indicted by their HPLC profile (Figure 2), obtained using the established method described in our previous reports [9, 21]. For successfully separating target compounds using CPC, a search for an optimum two-phase solvent system with a suitable partition coefficient (*K*) is required. The* K* value is the ratio of solute distributed between the mutually equilibrated two solvent phases, and the proper range of* K* values should be 0.5−2.0 [20]. The ratio of the two* K* values or the separation factor (*α* =* K*_1_/*K*_2_, where* K*_1_ >* K*_2_) provides useful information on resolution between two analytes and should be >1.5 [20]. As the combination of* n*-hexane-ethyl acetate-methanol-water (HEMWat) is the most widely used solvent system that provides a broad polarity range [20], a representative solvent system of* n*-hexane-ethyl acetate-methanol-water (5:5:5:5, v/v) was first tried for the separation of three monoterpenes. However, this solvent system provides extremely large* K* values (data not shown). Then, methanol was replaced by ethanol for their similar polarity and more appropriate *K* and *a* values could be given by *n*-hexane-ethyl acetate-ethanol-water (5:5:5:5, v/v). The results showed ethanol could be a good methanol substitute when HEMW at system could not provide good separation factor. Therefore, we tested different ratios of the two-phase solvent system (*n*-hexane-ethyl acetate-ethanol-water), and their measured* K* and *α* values are summarized in Table 1. When the two-phase solvent system comprising* n*-hexane-ethyl acetate-ethanol-water (5:5:5:5, v/v) in the descending mode was used, compounds** 1**-**3** could be well separated. However, compounds** 2** and** 3** could not be recovered from the aqueous mobile phase during the drying process due to their strong volatility. Therefore, an ascending mode using the upper organic phase as the mobile phase was applied to the separation of three compounds. According to the* K* and *α* values shown in Table 1, several solvent systems were tested. The results indicated that the biphasic solvent system of* n*-hexane-ethyl acetate-ethanol-water (8:2:8:2, v/v) in the ascending mode was suitable for separating three compounds, but the separation time for** 1** was extremely long. Therefore, based on the above results, the solvent system of* n*-hexane-ethyl acetate-ethanol-water (8:2:8:2, v/v) in the ascending mode was found to be satisfactory for the separation of** 2** and** 3** within short retention time, and the solvent system comprising* n*-hexane-ethyl acetate-ethanol-water (5:5:5:5, v/v) in the descending mode could be optimized the rapid isolation of** 1** with high purity.

### 3.2. CPC Separation and HPLC-DAD-ESI/MS Analysis of CPC Peak Fraction

**Figure 3:**
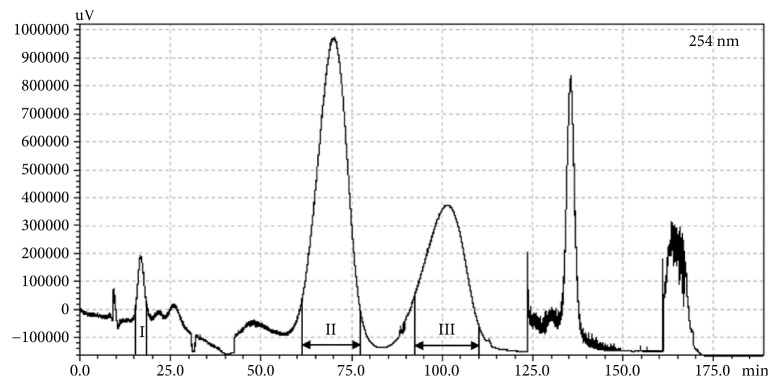
CPC separation of the SC-CO_2_ extract of the leaves of* P. frutescens* var.* crispa* (cv. Antisperill) using n-hexane/ethyl acetate/ethanol/water (5:5:5:5, v/v) in a descending mode (for CPC conditions, see Section 2). The extrusion was performed after 115 min.

**Figure 4:**
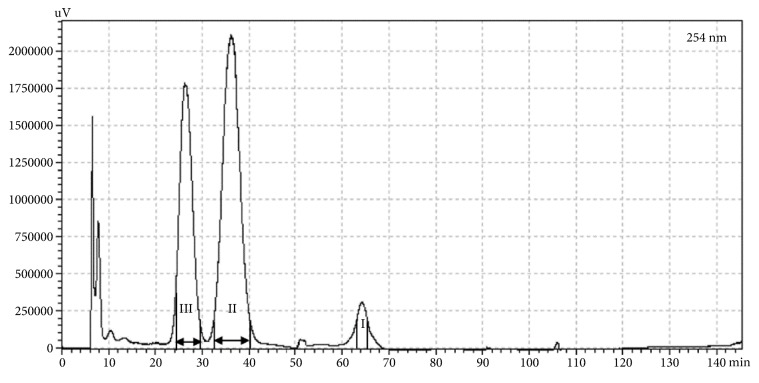
CPC separation of the SC-CO_2_ extract of the leaves of* P. frutescens* var.* crispa* (cv. Antisperill) using n-hexane/ethyl acetate/ethanol/water (8:2:8:2, v/v) in an ascending mode (for CPC conditions, see Section 2). The extrusion was performed after 42 min.

**Figure 5:**
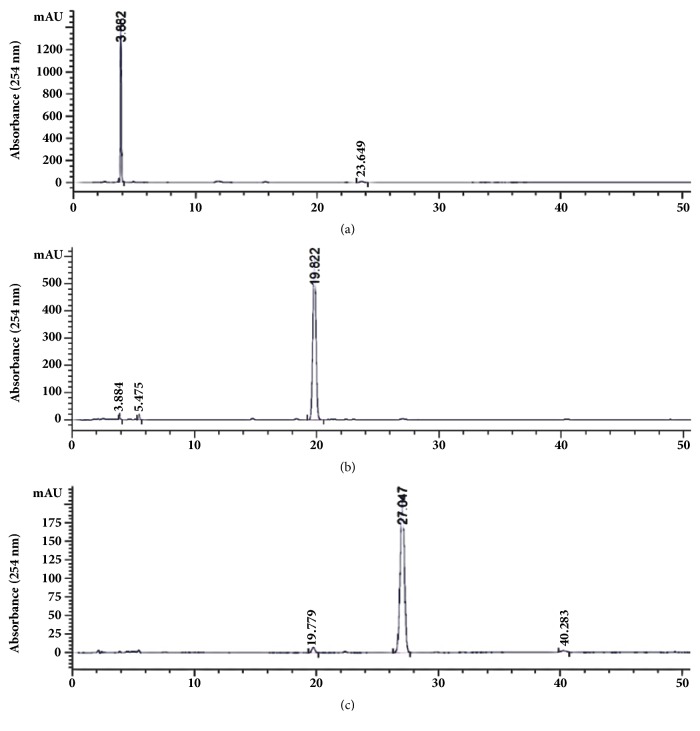
HPLC chromatograms of CPC peak fractions I (a), II (b), and III (c) (for chromatography conditions, see Section 2).

The SC-CO_2_ extract (500 mg) of the leaves of* P. frutescens* var.* crispa* (cv. Antisperill) was dissolved in a 1:1 (v/v) mixture (5 mL each) of the two-phase solvent system (*n*-hexane-ethyl acetate-ethanol-water = 5:5:5:5, v/v). The lower phase was used as the mobile phase in the descending mode. The retention of the stationary phase of this system was 70%. The peak fractions (I–III) were separated by CPC, and the separation time was approximately 110 min (Figure 3). Compound** 1** (2.4 mg) corresponding to the peak fraction I was obtained with 96.7% purity at 254 nm, however the volatile compounds** 2** and** 3** could not be obtained due to sample loss in the drying step. Therefore, CPC separation of compounds** 2** and** 3** was performed using the two-phase solvent system of *n*-hexane-ethyl acetate-ethanol-water (8:2:8:2, v/v) in the ascending mode (the upper organic mobile phase). In this solvent condition, the stationary phase retained in the column was 76%. After elution of the peaks III and II, extrusion of the stationary phase was performed at 42 min to reduce the retention time of peak I, which showed a high *K* value (Figure 4). Compounds** 2** (56.1 mg, 97.6% purity at 254 nm) and** 3** (78.6 mg, 96.1% purity at 254 nm) corresponding to the peak fractions II and III, respectively, were successfully purified from the SC-CO_2_ extract, however compound** 1** corresponding to the peak fraction I showed a very low purity of 76.2% (data not shown). The purity of the isolates was analysed by HPLC-DAD (Figure 5). The UV chromatograms and mass spectra of these compounds are provided in the Supplementary Materials (available here).

The preparative separation of compounds** 1** and** 2** using CCS was first reported in this study, although compound** 3** has already been separated by high speed countercurrent chromatography [19]. Compound** 1** was isolated as a new compound in our previous study [9], and compound** 2** has been reported to be isolated only from* Perilla* species [7, 22] and its synthesis has been studied [23], but a large-scale isolation of** 2** using CCS has not been reported before. Therefore, although the simultaneous separation of** 1** and** 2** was not possible in this study, the optimized conditions are developed for the rapid and large-scale purification of** 1** and** 2** respectively.

### 3.3. Structural Identification

The chemical structures of the target compounds were determined by ^1^H and ^13^C NMR spectroscopy and the NMR data are follows.

NMR data of peak I: positive ESI-MS* m/z* 181.2 [M+H]^+^. ^1^H-NMR (CDCl_3_, 500 MHz): *δ* 8.10 (1H, br s, H-5), 7.46 (1H, s, H-2), 7.08 (1H, d,* J *= 15.3 Hz, H-8), 6.84 (1H, br s, H-4), 6.77 (1H, d,* J* = 15.3 Hz, H-7), 1.40 (6H, s, H-10 and H-11); ^13^C-NMR (CDCl_3_, 125 MHz): *δ* 184.6 (C-6), 153.4 (C-8), 147.6 (C-5), 144.4 (C-2), 128.4 (C-3), 122.7 (C-7), 109.1 (C-4), 71.3 (C-9), and 29.6 (C-10 and C-11). Peak I was identified as 9-hydroxy isoegomaketone (**1**) [(2*E*)-1-(3-furanyl)-4-hydroxy-4-methyl-2-penten-1-one] on comparing its data with our previously obtained values [9].

NMR data of peak II: positive ESI-MS* m/z* 165.0 [M+H]^+^. ^1^H-NMR (CDCl_3_, 500 MHz): *δ* 8.03 (1H, s, H-5), 7.44 (1H, d,* J *= 1.5, H-2), 7.00 (1H, dd,* J *= 15.3, 1.5 Hz, H-8), 6.81 (1H, d,* J *= 1.5, H-4), 6.47 (1H, dd,* J *= 15.3, 1.5 Hz, H-7), 2.52 (1H, m, H-9), 1.10 (6H, s, 10 and H-11); ^13^C-NMR (CDCl_3_, 125 MHz): *δ* 184.2 (C-6), 154.6 (C-8), 147.2 (C-5), 147.2 (C-2), 129.3 (C-3), 124.1 (C-7), 109.2 (C-4), 31.3 (C-9), and 21.4 (C-10 and C-11). Peak II was identified as isoegomaketone (**2**) on comparing its data with the data provided elsewhere [21].

NMR data of peak III: positive ESI-MS* m/z* 167.1 [M+H]^+^. ^1^H-NMR (CDCl_3_, 500 MHz): *δ* 8.00 (1H, s, H-5), 7.40 (1H, d,* J *= 1.5, H-2), 6.73 (1H, d,* J *= 1.5, H-4), 2.70 (2H, t,* J* = 7.0 Hz, H-7), 1.58 (3H, m, H-8 and H-9), 0.89 (6H, d,* J *= 6.5, H-10 and H-11); ^13^C-NMR (CDCl_3_, 125 MHz): *δ* 195.7 (C-6), 147.7 (C-5), 144.3 (C-2), 128.2 (C-3), 109.1 (C-4), 38.9 (C-7), 33.6 (C-8), 28.2 (C-9), and 22.7 (C-10 and C-11). Peak III was identified as perilla ketone (**3**) on comparing its data with the data described elsewhere [21].

## 4. Conclusions

To the best of our knowledge, this is the first report that demonstrated the purification of 9-hydroxy isoegomaketone (**1**) and isoegomaketone (**2**) from* Perilla *species using CCS. Although these compounds with similar structures but different polarities were not simultaneously separated from the SC-CO_2_ extract of* P. frutescens* var.* crispa* (cv. Antisperill) by CPC, the adjustment of various solvent conditions and operation modes made it possible to optimize in a rapid and effective method for the preparative isolation of each compound. A two-phase solvent system comprising *n*-hexane-ethyl acetate-ethanol-water (5:5:5:5, v/v) in a descending mode was utilized in one-step purification of** 1**. And** 2** and perilla ketone (**3**) were successfully separated using the *n*-hexane-ethyl acetate-ethanol-water (8:2:8:2, v/v) solvent system in an ascending mode. Therefore, this study provides a reference for the large-scale isolation of monoterpenes at high purity from* Perilla *species

## Figures and Tables

**Table 1:** The partition coefficient (K) and separation factor (*α*) of compounds **1**–**3** in different solvent systems.

Solvent system (*n*-Hexane-EtOAc-EtOH-Water)	*K* values						*α* value		
	Descending mode			Ascending mode					
	**1**	**2**	**3**	**1**	**2**	**3**	*α*_12_	*α*_23_	*α*_13_
5:5:5:5	0.59	4.78	6.99	1.69	0.21	0.14	8.10	1.46	11.8
6:4:5:5	0.34	4.47	7.10	2.94	0.22	0.14	13.1	1.57	20.9
7:3:5:5	0.20	4.04	6.99	5.00	0.25	0.14	20.2	1.73	35.0
6:4:6:4	0.21	2.11	3.27	4.76	0.47	0.31	10.0	1.55	15.6
7:3:6:4	0.13	1.93	3.20	7.69	0.52	0.31	14.8	1.66	24.6
7:3:7:3	0.10	1.03	1.63	10.0	0.97	0.61	10.3	1.58	16.3
7:3:8:2	0.11	0.67	0.96	9.09	1.49	1.04	6.09	1.43	8.73
8:2:7:3	0.26	4.34	7.38	3.85	0.23	0.14	16.7	1.70	28.4
8:2:8:2	0.07	0.63	0.96	14.3	1.59	1.04	9.00	1.52	13.7
9:1:9:1	0.12	0.45	0.60	8.33	2.22	1.67	3.75	1.33	5.00

## Data Availability

The data supporting the findings of this study are available within the article and its Supplementary Materials. Raw data and additional information of this study are available from the corresponding author upon request.

## Supplementary Materials

Supplementary Materials As Supplementary Materials, Figures S1, S2, and S3 are included. Figure S1: UV and ESI-MS spectra of 9-hydroxy isoegomaketone (1) (for chromatography conditions, see Section 2). Figure S2: UV and ESI-MS spectra of isoegomaketone (2) (for chromatography conditions, see Section 2). Figure S3: UV and ESI-MS spectra of perilla ketone (3) (for chromatography conditions, see Section 2).
