# Supplementary material for: Preparative Separation of Three Monoterpenes from Perilla frutescens var. crispa Using Centrifugal Partition Chromatography
Source: Int J Anal Chem. 2019 Jan 9;2019:8751345. doi: 10.1155/2019/8751345 (PMC6343183; doi:10.1155/2019/8751345)
Supplement: Supplementary Materials — As Supplementary Materials, Figures S1, S2, and S3 are included. Figure S1: UV and ESI-MS spectra of 9-hydroxy isoegomaketone (1) (for chromatography conditions, see Section 2). Figure S2: UV and ESI-MS spectra of isoegomaketone (2) (for chromatography conditions, see Section 2). Figure S3: UV and ESI-MS spectra of perilla ketone (3) (for chromatography conditions, see Section 2). [file 8751345.f1.docx]

**SUPPLEMENTARY MATERIALS**

**Preparative separation of three monoterpenes from *Perilla frutescens* var. *crispa* using centrifugal partition chromatography**

Bomi Nam,^1,4^ Sunil Babu Paudel,^2,4^ Jin-Baek Kim,^1^ Chang Hyun Jin,^1^ Dongho Lee,^3^ Joo-Won Nam,^2^ Ah-Reum Han^1^

*^1^Advanced Radiation Technology Institute, Korea Atomic Energy Research Institute, Jeongeup-si, Jeollabuk-do 56212, Republic of Korea*

*^2^College of Pharmacy, Yeungnam University, Gyeongsan-si, Gyeongsangbuk-do 38541, Republic of Korea*

*^3^Department of Biosystems and Biotechnology, Korea University, Seoul 02841, Republic of Korea*

*^4^These authors contributed equally to this work.*

Correspondence should be addressed to Ah-Reum Han; arhan@kaeri.re.kr and Joo-Won Nam; jwnam@yu.ac.kr

Figure S1: UV and ESI-MS spectra of 9-hydroxy isoegomaketone (**1**) (for chromatography conditions, see Section 2).

Figure S2: UV and ESI-MS spectra of isoegomaketone (**2**) (for chromatography conditions, see Section 2).

Figure S3: UV and ESI-MS spectra of perilla ketone (**3**) (for chromatography conditions, see Section 2).
